# De Garengeot Hernia, an acute appendicitis in the right femoral hernia canal, and successful management with transabdominal closure and appendectomy: a case Report

**DOI:** 10.1186/s12894-023-01383-7

**Published:** 2024-02-16

**Authors:** Po-Chuan Yu, Ling-Ting Wang, Chun-Yu Chang, Yao-Chou Tsai, Kian-Hwee Chong

**Affiliations:** 1 Department of Anesthesiology, Taipei Tzu Chi Hospital, Buddhist Tzu Chi Medical Foundation, New Taipei City, 23142 Taiwan; 2 Department of Urology, Taipei Tzu Chi Hospital, Buddhist Tzu Chi Medical Foundation, New Taipei City, 23142 Taiwan; 3 Department of Surgery, Taipei Tzu Chi Hospital, Buddhist Tzu Chi Medical Foundation, New Taipei City, 23142 Taiwan; 4 Department of Surgery, School of Medicine, Buddhist Tzu Chi University, Hualien, 97004 Taiwan; 5 Department of General Surgery, Taipei Tzu Chi Hospital, No.289, Jianguo Rd., Xindian Dist, New Taipei, 231405 Taiwan

**Keywords:** De Garengeot hernia, Amyand’s hernia, Femoral hernia, Appendicitis, Appendectomy, Hernioplasty, Transversus Abdominis plane block

## Abstract

Incarceration of the appendix within a femoral hernia is a rare condition of abdominal wall hernia about 0.1 to 0.5% in reported femoral hernia [1]. We report a case of a 56-year-old female whose appendix was trapped in the right femoral canal. There are few reports in the literature on entrapment of the appendix within a femoral hernia. The management of this condition includes antibiotics, drainage appendectomy, hernioplasty and mesh repair.

**Supplementary Information:**

The online version contains supplementary material available at 10.1186/s12894-023-01383-7.

## Introduction

Femoral hernias constitute a minority of abdominal wall hernias and are more common in women [2]. An entrapment of the appendix into the femoral hernia is called a de Garengeot hernia, which was named after Rene Jacques Croissant de Garengeot (1688–1759), a French surgeon, who first reported this pathology in 1731 [3].

Amyand’s hernia is another rare hernia that the appendix entrapped in the inguinal sac and predominance in man and should not be confused with this hernia (Table 1). In 1735, Claudius Amyand (1660–1735), a surgeon in England, performed the first successful appendectomy on an 11-year-old boy with a perforated, acutely inflamed appendix within the right scrotal sac [4].

**Table 1:** Comparison of De Garengeot Hernia and Amyand’s Hernia

	De Garengeot hernia	Amyand’s Hernia
Orifice	Femoral canal	Inguinal canal
Incidence	0.1-5% of femoral hernia	1% of inguinal hernia
Sex	Female	Male

Clinical experience and a literature review provided limited information on this problem and its management. There were about one hundred cases in a search on Pubmed. There was only an analysis of this problem, which reported that clinical presentation, laboratory and plain radiological investigations might not facilitate the diagnosis and surgical approach was also unconcluded. [5] and a review article concluded a classification [6]. The mesh placement is under debate [1, 5–7].

We herein report a patient with an inflamed appendix causing by incarcerated right femoral hernia and successful management with laparoscopic appendectomy and transabdominal closure with suture.

## Case report

**Fig. 1:**
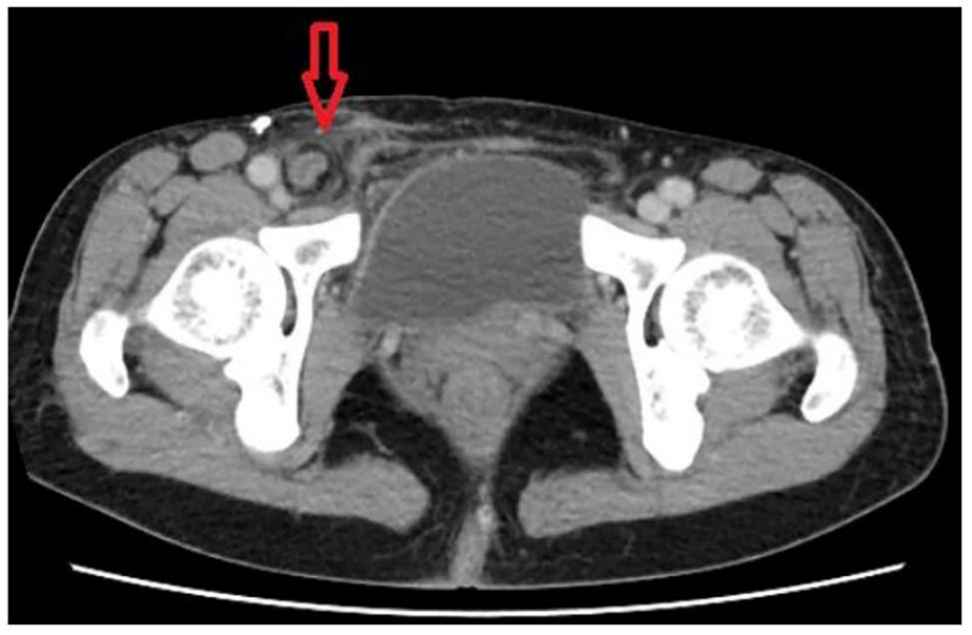
Axial plane of multidetector computed tomography image. A dilated appendix entrapped in femoral canal and some fluid collection

**Fig. 2:**
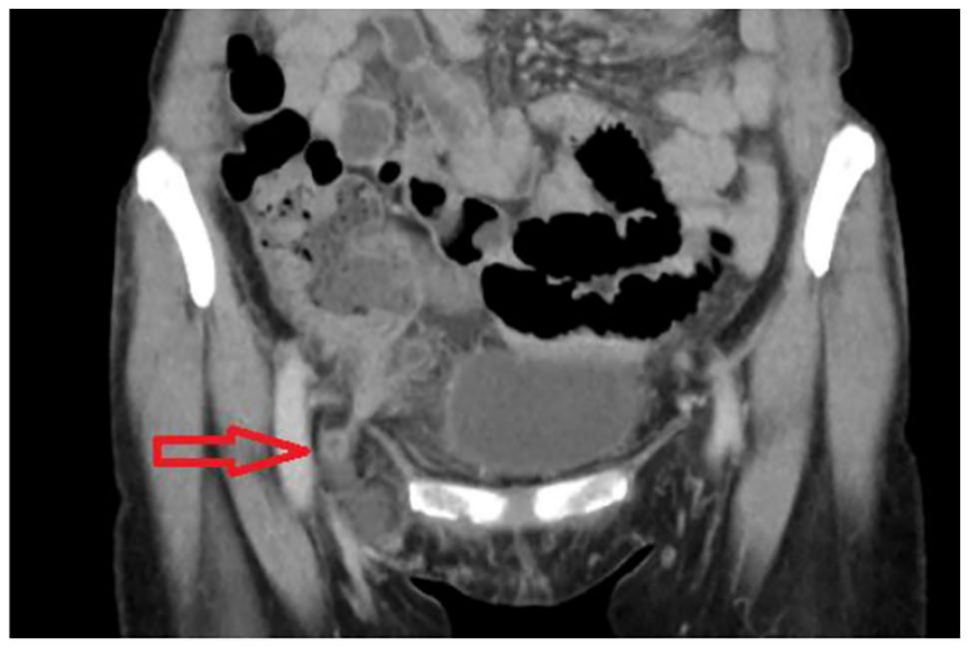
Coronal plane of multidetector computed tomography image. Another plane of entrapped appendix and fat stranding at right lower abdomen

A 56-year-old woman presented to the emergency department with a 3-day history of dull pain over the right lower quadrant which was shifted from peri-umbilicus initially associated with a bulge mass in the right groin area which was not enlarged with cough, standing or weight-bearing. She had undergone laparoscopic complete extraperitoneal inguinal hernia repair 3 years previously in other hospital. According to her statement, there was a mesh placement on right side and simple repair at left side. She denied other medical diseases except uterine myoma about 2 to 3 cm under regular follow-up at gynecology. Her vital signs were normal, and she was afebrile. On physical examination, there was a right-groin protruding mass below the inguinal line and a positive Mcburney’s sign. Laboratory data demonstrated leukocytosis (10.12*10^3/µL, normal range 6 ~ 10*10^3/µL) with neutrophil predominance (83.3%). Abdominal computed tomography showed a right femoral hernia incarcerated by appendix with appendix dilatation (8.9 mm), fat stranding and some fluid collection in the right inguinal region (Figs. 1 and 2).

**Fig. 3:**
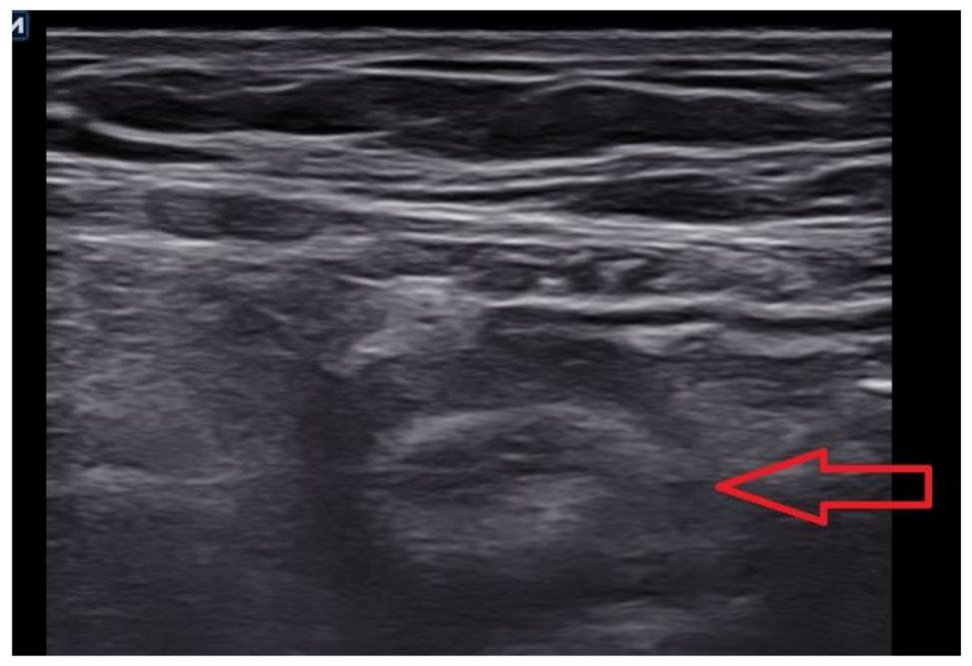
Round, aperistaltic, noncompressible and dilated appendix; distinct appendiceal wall layers; periappendiceal fluid collection; and wall thickening on sonography

**Fig. 4:**
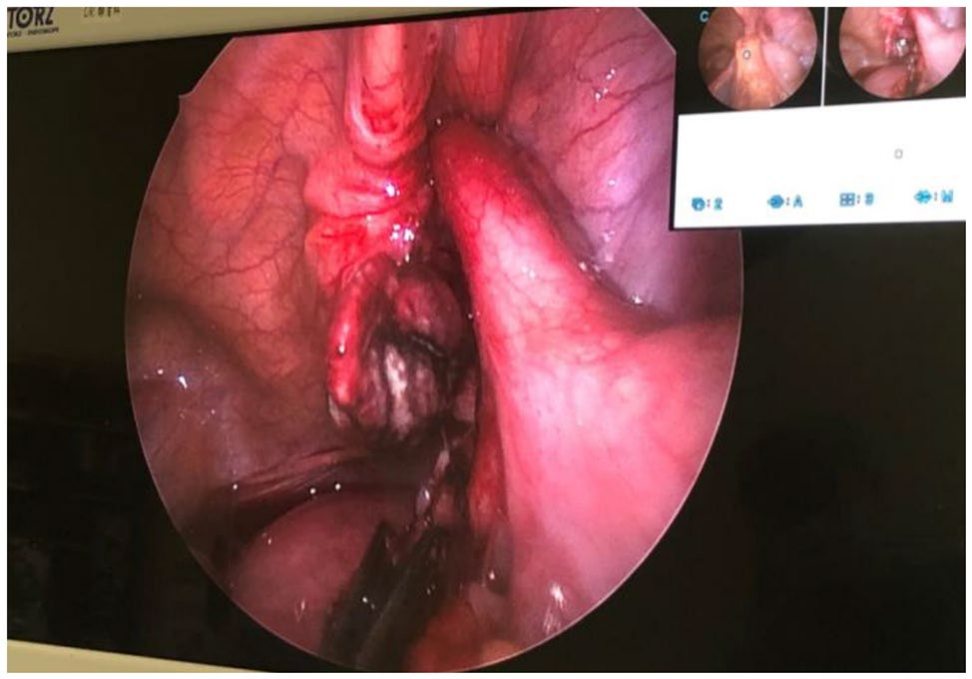
View of laparoscopy. Middle appendix incarcerated inner ring of femoral canal

The patient received endotracheal general anesthesia and a transversus abdominis plane block after discussion with anesthesiologist at anesthesia assessment. The sonography findings included a round, aperistaltic, noncompressible and dilated appendix, distinct appendiceal wall layers, periappendiceal fluid collection and wall thickening (Fig. 3). During laparoscopy, the distal part of appendix is gangrenous and middle part of appendix incarcerated inner ring of right femoral canal (Fig. 4), and left inguinal area was also checked without abvious finding Appendectomy was performed after reduction. The right femoral peritoneum of the internal ring was closed with a primary suture. The pathology revealed acute suppurative appendicitis with acute serositis. Our patient was treated with antibiotics, flomoxef, and drainage of the abdominal fluid collection. The analgesics were prescribed orally and she only took twice pills during hospitalization. She felt better than previous TEP generally. She started normal diet per os on postoperative day 1. There was no significant abdominal fluid drainage. There was no abdominal symptom such as pain or nausea. The laboratory data revealed no abvious inflammation by decreased number of white blood cells and level of C-reactive protein. She was discharged 4 days after successful appendectomy and hernia repair. She was seen at the surgical outpatient clinic 2 weeks later with a clean, well-healed surgical scar. There was no erythema at groin area or protruding mass at low abdomen.

## Discussion

Femoral hernia is a less common type of abdominal wall hernia [1, 2, 7]. Protrusion of the appendix in the femoral hernia area is called a de Garengeot hernia. In 1731, Rene Jacques Croissant de Garengeot, a French surgeon, first reported this pathology in a 55-year-old woman who developed this disease after lifting weights [3].

The incidence of De Garengeot hernia is approximately 0.1–5% of reported femoral hernias and 0.1% of acute appendicitis [1]. De Garengeot hernia is more common, as anticipated, in women compared with Amyand’s hernia, which is more often seen in men [8] (Table 1). Patients with a history of inguinal hernia repair are more prone to De Garengeot hernia [9]. This hernia may be incarcerated by completely healthy, inflamed, infected, unruptured, ruptured, or incarcerated appendix [5, 6]. The chief complaints related to this disease include groin tenderness combined with a tender irreducible bulge in the femoral area [5]. Groin erythema was seen in 33% of patients and may indicate a ruptured appendix or abscess [6]. Other presentations may be fever, vomiting, and intestinal obstruction according to the condition of the appendix (healthy, inflamed, infected, unruptured, ruptured, or incarcerated). Abdominal pain was a minor complaint. In some ruptured cases, there is still a lack of abdominal symptoms, which may be explained by the restricted space of the femoral canal. These symptoms make the diagnosis of De Garengeot hernia difficult, as it mimics incarcerated hernia. Abdominal examination, laboratory tests and plain roentgenograms do not aid the differential diagnosis [1, 5, 6, 8]. Abdominal computed tomography (CT) is helpful for diagnosing acute abdominal pain. Takemuraet al. first reported a De Garengeot hernia before surgery on CT [10], which provides a direct view of the appendix in the femoral area. Sonography was also useful for making this diagnosis. The hernia appears as a blind-ended tubular structure with thickened walls on sonography [8].

Losanoff reported a classification system for Amyand’s hernia according to the status of the appendix, and intra-abdominal pathology and has been proposed to guide treatment [11]. Amyand’s hernia classification may not be useful for De Garengeot hernia because the opening of the femoral canal is smaller than the inguinal sac [12] and prevents the spread of infection. Guenther et al. [6] described a classification (Table 2) for De Garengeot hernia and may be used for the surgical management of De Garengeot hernia.

**Table 2:** Classification of De Garengeot Hernia by Guenther et al. [6]

Class	Description
1	Normal appendix
2	
2A	Appendix with inflammation, erythema, or congestion
2B	2A and erythema of the cecum or other part of the large or small intestine
3	
3A	Appendix with isolated necrosis at the tip
3B	Whole appendix with necrosis
4	Appendix with necrosis and necrosis of the cecum or other part of the large or small intestine
5	Appendix with rupture, abscess, or fistula

Although open surgery is considered the standard procedure in emergency conditions, there have been several reports regarding the laparoscopic approach [7, 13]. When a ruptured appendix and abscess are seen on CT and local erythema is present, the laparoscopic approach may provide inadequate infection control in the groin [6]. Guenther et al. [6] recommended that cases classified as Class 2 or higher should undergo appendectomy. Mesh repair of femoral hernia is associated with a lower recurrence rate than that related to simple repair [14]; thus, mesh placement should be considered to lower the risk of contaminating the field [5, 6].

In our patient, her inflamed appendix entrapped in femoral canal not in inguinal sac, typical type of De Garengeot hernia not Amyand’s hernia. Her risk factors such as female, previous same side hernia repair were comparable to our literature review. Our patient has no groin tenderness or erythema which indicated unrupture or not severe inflamed appendix. According to Guenther’s classification, she had De Garengeot hernia class 2B due to inflamed appendix and terminal ileum [6]. Appendectomy is recommended but mesh repair is unconcluded while most surgeons preceded primary suture via open repair. Because of previous left inguinal hernia and unruptured appendix, we used transabdominal approach which is able to exam the rest of the abdomen.

Though there was low risk of mesh infection at class 2 A and improvement of aseptic technique, surgical technique, the peri- and post-operative care of patients and easy utility of advanced diagnosis tool such as CT and sonography, mesh placement may consider using in higher classification after shared decision-making about infection and further hernia recurrence.

## Conclusion

A right femoral hernia caused by an inflamed appendix is relatively rare. It is important for physicians to carefully examine the appendix before appendectomy and estimate the necessity of mesh repair depending on infection status of the vermiform appendix. Understanding this unusual femoral hernia may facilitate further clinical thinking about surgical approach and mesh placement in the future.

### Electronic supplementary material

Below is the link to the electronic supplementary material.

Supplementary Material 1

## Data Availability

All data and figure generated or analyzed during this study are included in this published article and its supplementary information files.

## References

[1] Bidarmaghz B, Tee CL. *A case of De Garengeot hernia and literature review* BMJ Case Rep, 2017. 2017. 10.1136/bcr-2017-220926 PMC5589054 28882935

[2] Shakil A (2020). Inguinal hernias: diagnosis and management. Am Fam Physician.

[3] RJC DG. *Traite des operations de chirurgie* 2nd edn. Paris, 1731: p. 369–371.

[4] Claude A. *Of an inguinal rupture, with a pin in the appendix coeci, incrusted with stone; and some observations on wounds in the guts*. Phil Trans R Soc, 1736. 39(443): p. 329–42.

[5] Linder S, Linder G, Mansson C (2019). Treatment of De Garengeot’s hernia: a meta-analysis. Hernia.

[6] Guenther TM (2021). De Garengeot hernia: a systematic review. Surg Endosc.

[7] Comman A (2007). DeGarengeot hernia: transabdominal preperitoneal hernia repair and appendectomy. JSLS.

[8] Abdulghaffar S (2019). CT and ultrasound findings in a case of De Garengeot’s hernia: a case report. Radiol Case Rep.

[9] Mikkelsen T, Bay-Nielsen M, Kehlet H (2002). Risk of femoral hernia after inguinal herniorrhaphy. Br J Surg.

[10] Takemura M, Goshi IK, Osugi S, Kinoshita H (2000). Strangulated femoral hernia containing gangrenous Appendicitis: report of a case. Nihon Gekakei Rengo Gakkaishi.

[11] Losanoff JE, Basson MD (2007). Amyand hernia: what lies beneath–a proposed classification scheme to determine management. Am Surg.

[12] Yoong P, Duffy S, Marshall TJ (2013). The inguinal and femoral canals: a practical step-by-step approach to accurate sonographic assessment. Indian J Radiol Imaging.

[13] Hachisuka T (2003). Femoral hernia repair. Surg Clin North Am.

[14] George A, Sarosi J, MDKfir Ben-David MD. FACS. *Recurrent inguinal and femoral hernia*. 2022; Available from: https://www.uptodate.com/contents/recurrent-inguinal-and-femoral-hernia.

