# Supplementary material for: De Garengeot Hernia, an acute appendicitis in the right femoral hernia canal, and successful management with transabdominal closure and appendectomy: a case Report
Source: BMC Urol. 2024 Feb 16;24:41. doi: 10.1186/s12894-023-01383-7 (PMC10870586; doi:10.1186/s12894-023-01383-7)
Supplement: Supplementary file 1 — Supplementary Material 1 [file 12894_2023_1383_MOESM1_ESM.docx]

**Supplementary file:**

Axial plane of multidetector computed tomography images

| Saved Image0070 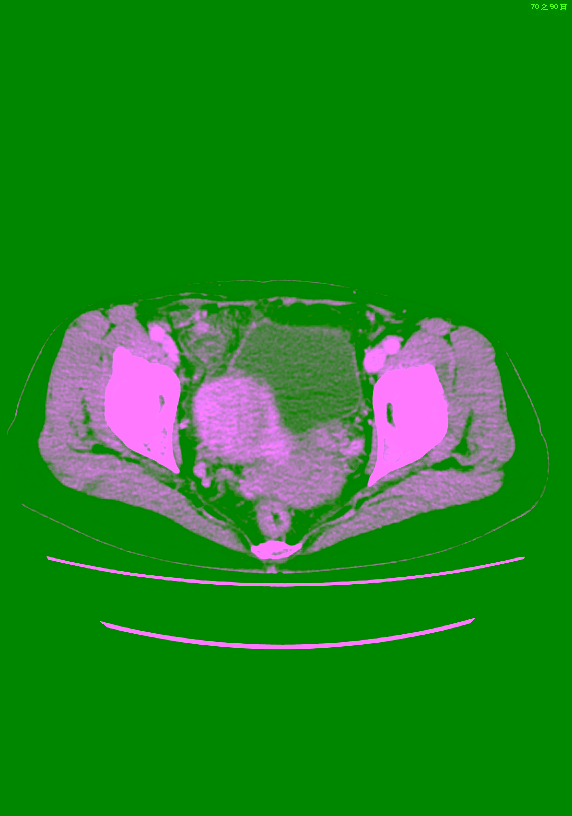 | Saved Image0074  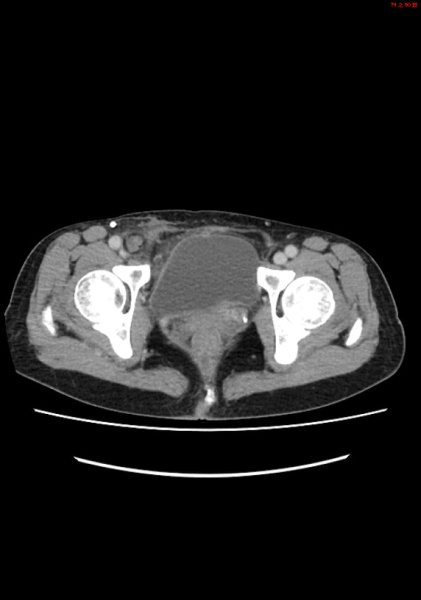 | Saved Image0078  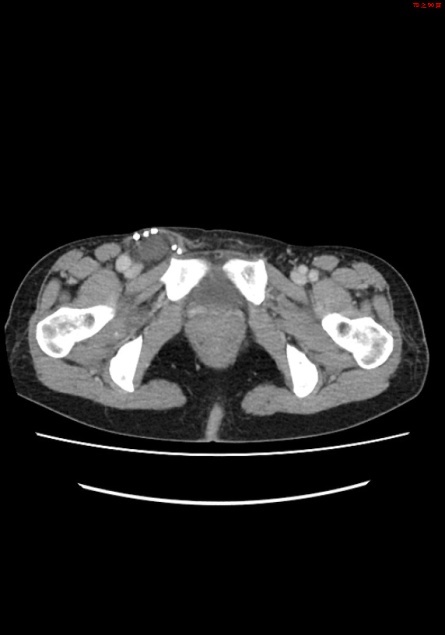 |
| --- | --- | --- |
| 1. An appendix with dilatation and fat stranding | 1. A right femoral hernia with appendix herniation causing appendix dilatation | 1. Clips of previous hernia repair |

Coronal plane of multidetector computed tomography images

| Saved Image0005  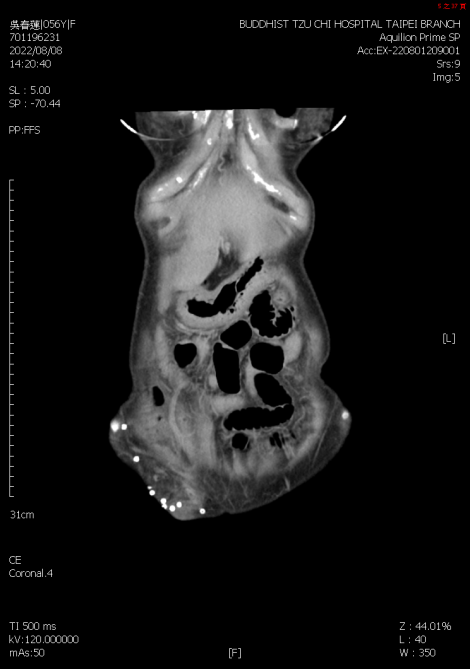 | Saved Image0008  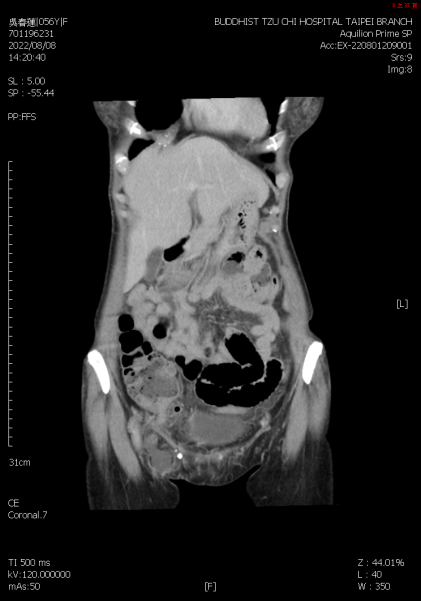 | Saved Image0010  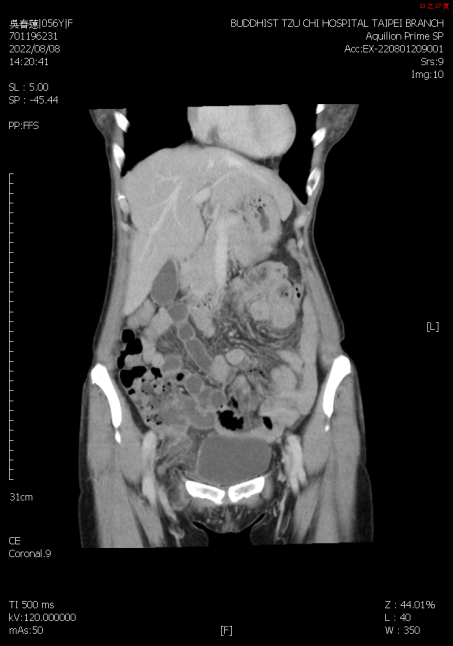 |
| --- | --- | --- |
| 1. Clips of previous hernia repair | 1. An appendix herniation below the inguinal ligament | 1. A right femoral hernia with appendix herniation |
